# Vector Generation of Quantum Contextual Sets in Even Dimensional Hilbert Spaces

**DOI:** 10.3390/e20120928

**Published:** 2018-12-05

**Authors:** Mladen Pavičić, Norman D. Megill

**Affiliations:** 1 Nano Optics, Department of Physics, Humboldt University, 12489 Berlin, Germany; 2 Center of Excellence for Advanced Materials and Sensors, Research Unit Photonics and Quantum Optics, Institute Ruder Bošković, 10000 Zagreb, Croatia; 3 Boston Information Group, Lexington, MA 02420, USA

**Keywords:** quantum contextuality, Kochen–Specker sets, MMP hypergraphs, Greechie diagrams

## Abstract

Recently, quantum contextuality has been proved to be the source of quantum computation’s power. That, together with multiple recent contextual experiments, prompts improving the methods of generation of contextual sets and finding their features. The most elaborated contextual sets, which offer blueprints for contextual experiments and computational gates, are the Kochen–Specker (KS) sets. In this paper, we show a method of vector generation that supersedes previous methods. It is implemented by means of algorithms and programs that generate hypergraphs embodying the Kochen–Specker property and that are designed to be carried out on supercomputers. We show that vector component generation of KS hypergraphs exhausts all possible vectors that can be constructed from chosen vector components, in contrast to previous studies that used incomplete lists of vectors and therefore missed a majority of hypergraphs. Consequently, this unified method is far more efficient for generations of KS sets and their implementation in quantum computation and quantum communication. Several new KS classes and their features have been found and are elaborated on in the paper. Greechie diagrams are discussed.

## 1. Introduction

Recently, it has been discovered that quantum contextuality might have a significant place in a development quantum communication [1,2], quantum computation [3,4], and lattice theory [5,6]. This has prompted experimental implementation of 4-, 6-, and 8-dimensional contextual experiments with photons [7,8,9,10,11,12,13], neutrons [14,15,16], trapped ions [17], solid state molecular nuclear spins [18], and paths [19,20].

Experimental contextual tests involve subtle issues, such as the possibility of noncontextual hidden variable models that can reproduce quantum mechanical predictions up to arbitrary precision [21]. These models are important because they show how assignments of predetermined values to dense sets of projection operators are precluded by any quantum model. Thus, Spekkens [22] introduces generalised noncontextuality in an attempt to make precise the distinction between classical and quantum theories, distinguishing the notions of preparation, transformation, and measurement of noncontextuality and by doing so demonstrates that even the 2D Hilbert space is not inherently noncontextual. Kunjwal and Spekkens [23] derive an inequality that does not assume that the value assignments are deterministic, showing that noncontextuality cannot be salvaged by abandoning determinism. Kunjwal [24] shows how to compute a noncontextuality inequality from an invariant derived from a contextual set/configuration representing an experimental Kochen-Specker (KS) setup. This opens up the possibility of finding contextual sets that provide the best noise robustness in demonstrating contextuality. The large number of such sets that we show in the present work can provide a rich source for such an effort.

Quantum contextual configurations that have been elaborated on the most in the literature are the KS sets, and, in this paper, we consider just them. In order to obtain KS sets, so far, various methods of exploiting correlations, symmetries, geometry, qubit states, Pauli states, Lie algebras, etc., have been found and used for generating master sets i.e., big sets which contain all smaller contextual sets [25,26,27,28,29,30,31,32,33,34,35,36,37].

All of these methods boil down either to finding a list of vectors and their *n*-tuples of orthogonalities from which a master set can be read off or finding a structure, e.g., a polytope, from which again a list of vectors and orthogonalities can be read off as well as a master set they build. In the present paper, we take the simplest possible vector components within an *n*-dimensional Hilbert space, e.g., {0,±1}, and via our algorithms and programs exhaustively build all possible vectors and their orthogonal *n*-tuples and then filter out KS sets from the sets in which the vectors are organized. For a particular choice of components, the chances of getting KS sets are very high. We generate KS sets for even-dimensional spaces, up to 32, that properly contain all previously obtained and known KS sets, present their features and distributions, give examples of previously unknown sets, and present a blueprint for implementation of a simple set with a complex coordinatization.

## 2. Results

The main results presented in this paper concern generation of contextual sets from several basic vector components. Previous contextual sets from the literature made use of often complicated sets of vectors that the authors arrived at, following particular symmetries, or geometries, or polytope correlations, or Pauli operators, or qubit states, etc. In contrast, our approach considers McKay–Megill–Pavičić (MMP) hypergraphs (defined in Section 2.1) from *n*-dimensional (*n*D) Hilbert space ($H^{n}$, n≥3) originally consisting of *n*-tuples (in our approach represented by MMP hypergraph edges) of orthogonal vectors (MMP hypergraph vertices) which exhaust themselves in forming configurations/sets of vectors (MMP hypergraphs). Already in [38], we realised that hypergraphs massively generated by their non-isomorphic upward construction might satisfy the Kochen–Specker theorem even when there were no vectors by means of which they might be represented (see Theorem 1), and finding coordinatizations for those hypergraphs which might have them, via standard methods of solving systems of non-linear equations, is an exponentially complex task solvable only for the smallest hypergraphs [38]. It was, therefore, rather surprising to us to discover that the hypergraphs formed by very simple vector components often satisfied the Kochen–Specker theorem. In this paper, we present a method of generation of KS MMP hypergraphs, also called KS hypergraphs, via such simple sets of vector components.

**Theorem** **1** (MMP hypergraph reformulation of the Kochen–Specker theorem)**.**  

*There are n*D MMP *hypergraphs, i.e., those whose each edge contains n vertices, called* KS MMP *hypergraphs, to which it is impossible to assign 1s and 0s in such a way that*

*(α)*  No two vertices within any of its edges are both assigned the value 1;
*(β)*  In any of its edges, not all of the vertices are assigned the value 0.

**Figure 1:**
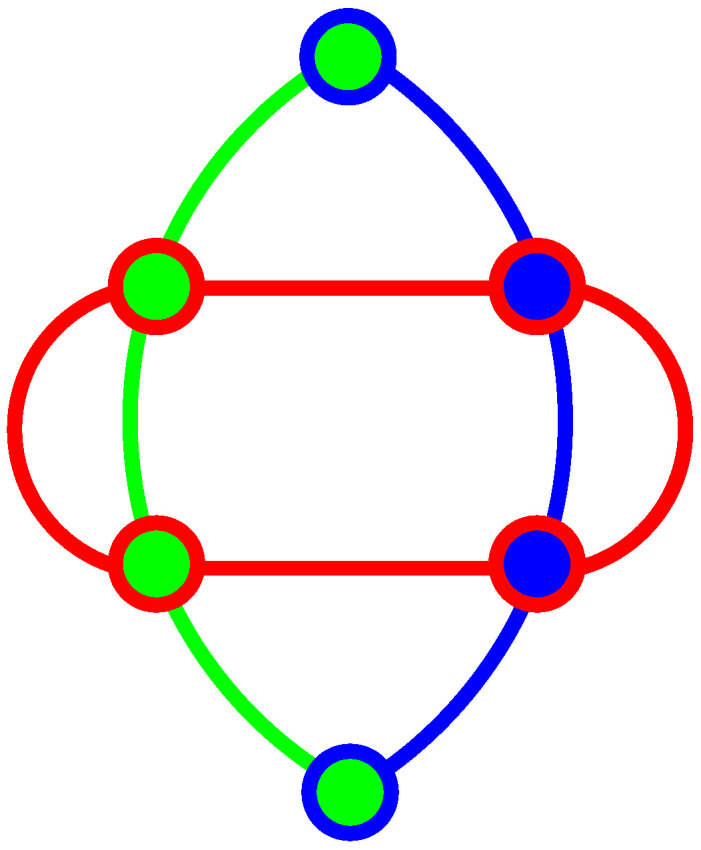
The smallest 4D KS MMP hypergraph without a coordinatization.

In Figure 1, we show the smallest possible 4D KS MMP hypergraph with six vertices and three edges. We can easily verify that it is impossible to assign 1 and 0 to its vertices so as to satisfy the conditions (α) and (β) from Theorem 1. For instance, if we assign 1 to the top green-blue vertex, then, according to the condition (α), all of the other vertices contained in the blue and green edges must be assigned value 0, but, herewith, all four vertices in the red edge are assigned 0s in violation of the condition (β), or, if we assign 1 to the top red-blue vertex, then, according to the condition (α), all the other vertices contained in the blue and red edges must be assigned value 0, but, herewith, all four vertices in the green edge are assigned 0s in violation of the condition (β). Analogous verifications go through for the remaining four vertices. We verified that there is neither a real nor complex vector solution of a corresponding system of nonlinear equations [38]. We have not tried quaternions as of yet.

When a coordinatization of a KS MMP hypergraph exists, its vertices denote *n*-dimensional vectors in $H^{n}$, n≥3, and edges designate orthogonal *n*-tuples of vectors containing the corresponding vertices. In our present approach, a coordinatization is automatically assigned to each hypergraph by the very procedure of its generation from the basic vector components. A KS MMP hypergraph with a given coordinatization of whatever origin we often simply call a KS *set*.

### 2.1. Formalism

MMP hypergraphs are those whose edges (of size *n*) intersect each other in at most n−2 vertices [26,37]. They are encoded by means of printable ASCII characters. Vertices are denoted by one of the following characters: 1 2 ...9 A B ...Z a b ...z ! " # $ % & ’ ( ) * - / : ; < = > ? @ [ ∖ ] _ ‘ { | } ~ [26]. When all of them are exhausted, one reuses them prefixed by ‘+’, then again by ‘++’, and so forth. An *n*-dimensional KS set with *k* vectors and *m n*-tuples is represented by an MMP hypergraph with *k* vertices and *m* edges which we denote as a *k*-*m* set. In its graphical representation, vertices are depicted as dots and edges as straight or curved lines connecting *m* orthogonal vertices. We handle MMP hypergraphs by means of algorithms in the programs SHORTD, MMPSTRIP, MMPSUBGRAPH, VECFIND, STATES01, and others [5,30,38,39,40,41]. In its numerical representation (used for computer processing), each MMP hypergraph is encoded in a single line in which all *m* edges are successively given, separated by commas, and followed by assignments of coordinatization to *k* vertices (see 18-9 in Section 2.2).

### 2.2. KS Vector Lists vs. Vector Component MMP Hypergraphs

In Table 1, we give an overview of most of the *k*-*m* KS sets (KS hypergraphs with *m* vertices and *k* edges) as defined via lists and tables of vectors used to build the KS master sets that one can find in the literature. These master sets serve us to obtain billions of non-isomorphic smaller KS sets (KS subsets, subhypergraphs) which define *k*-*m classes*. In doing so (via the aforementioned algorithms and programs), we keep to minimal, *critical*, KS subhypergraphs in the sense that a removal of any of their edges turns them into non-KS sets. Critical KS hypergraphs are all we need for an experimental implementation: additional orthogonalities that bigger KS sets (containing critical ones) might possess do not add any new property to the ones that the minimal critical core already has. The smallest hypergraphs we give in the table are therefore the smallest criticals. Many more of them, as well as their distributions, the reader can find in the cited references. Some coordinatizations are over-complicated in the original literature. For example (as shown in [37]), for the 4D 148-265 master, components $\left\{0,\pm i,\pm 1,\pm \omega ,\pm \omega^{2}\right\}$, where $\omega =e^{2\pi i/3}$, suffice for building the coordinatization, and for the 6D 21-7 components {0,1,ω} suffice. In addition, {0,±1} suffice for building the 6D 236-1216.

Some of the smallest KS hypergraphs in the table have ASCII characters assigned and some do not. This is to stress that we can assign them in an arbitrary and random way to any hypergraph and then the program VECFIND will provide them with a coordinatization in a fraction of a second. For instance,

**18-9**: 1234,4567,789A,ABCD,DEFG,GHI1,I29B,35CE,68FH.

{1={0,0,0,1},2={0,0,1,0},3={1,1,0,0},4={1,-1,0,0},5={0,0,1,1},6={1,1,1,-1},

7={1,1,-1,1}, 8={1,-1,1,1},9={1,0,0,-1},A={0,1,1,0},B={1,0,0,1},C={1,-1,1,-1},

D={1,1,-1,-1},E={1,-1,-1,1},F={0,1,0,1},G={1,0,1,0},H={1,0,-1,0},I={0,1,0,0}}.

(To simplify parsing, this notation delineates vectors with braces instead of traditional parentheses in order to reserve parentheses for component expressions.)

However, a real finding is that we can go the other way round and determine the KS sets from nothing but vector components {0,±1}.

### 2.3. Vector-Component-Generated Hypergraph Masters

We put simplest possible vector components, which might build vectors and therefore provide a coordinatization to MMP hypergraphs, into our program VECFIND. Via its option -master, the program builds an internal list of all possible non-zero vectors containing these components. From this list, it finds all possible edges of the hypergraph, which it then generates. MMPSTRIP via its option -U separates unconnected MMP subgraphs. We pipe the obtained hypergraphs through the program STATES01 to keep those that possess the KS property. We can use other programs of ours, MMPSTRIP, MMPSHUFFLE, SHORTD, STATES01, LOOP, etc., to obtain smaller KS subsets and analyze their features.

The likelihood that chosen components will give us a KS master hypergraph and the speed with which it does so depends on particular features they possess. Here, we will elaborate on some of them and give a few examples. Features are based on statistics obtained in the process of generating hypergraphs:

(*i*) the input set of components for generating two-qubit KS hypergraphs (4D) should contain number pairs of opposite signs, e.g., ±1, and zero (0); we conjecture that the same holds for 3, 4, ...qubits; with 6D it does not hold literally; e.g., {0,1,ω} generate a KS master; however, the following combination of ω’s gives the opposite sign to 1: $\omega +\omega^{2}=-1$;
(*ii*) mixing real and complex components gives a denser distribution of smaller KS hypergraphs;
(*iii*) reducing the number of components shortens the time needed to generate smaller hypergraphs and apparently does not affect their distribution.

Feature (*i*) means that, no matter how many different numbers we use as our input components, we will not get a KS master if at least to one of the numbers, the same number with the opposite sign is not added. Thus, e.g., {0,1,−i,2,−3,4,5} or a similar string does not give any, while {0,±1}, or {0,±i}, or $\left\{0,\pm \left(\sqrt{5}-1\right)/2\right\}$ do. Of course, the latter strings all give mutually isomorphic KS masters, i.e., one and the same KS master, if used alone. More specifically, they yield a 40-32 master with 40 vertices and 32 edges as shown in Table 2. When any of them are used together with other components, although they would generate different component-masters, all the latter masters of a particular dimension would have a common smallest hypergraph as also shown in Table 2.

We obtained the following particular results which show the extent to which component-masters give a more populated distribution of KS criticals than list-masters. We also closed several open questions:

- As for the features (ii) and (iii) above, components {0,±1,ω} generate the master 180-203 which has the following smallest criticals 18-9, 20...22-11, 22...26-13, 24...30-15, 30...31-16, 28...35-17, 33...37-18, etc. This distribution is much denser than that of, e.g., the list-master 24-24 with real vectors which in the same span of edges consists only of 18-9, 20-11, 22-13, and 24-15 criticals or of the list-master 60-75 which starts with the 26-13 critical. In Appendix A, we give a detailed description of a 21-11 critical with a complex coordinatization and give a blueprint for its experimental implementation;
- In [19], the reader is challenged to find a master set which would contain the "seven context star" 21-7 KS critical (shown in Table 1 and Table 2). We find that {0,1,ω} generate the 216-153 6D master which contains just three criticals 21-7, 27-9, and 33-11, $\left\{0,1,\omega ,\omega^{2}\right\}$ generate 834-1609 master from which we obtained $2.5\times 10^{7}$ criticals, and $\left\{0,\pm 1,\omega ,\omega^{2}\right\}$ generate 11808-314446 master from which we obtained $3\times 10^{7}$ criticals, all of them containing the seven context star. Some of the obtained criticals are given in Appendix B;
- The 60-75 list-master contains criticals with up to 41 edges and 60 vertices, while the 2316-3052 component-master generated from the same vector components contains criticals with up to close to 200 edges and 300 vertices;
- The 60-105 list-master contains criticals with up to 40 edges and 60 vertices, while the 156-249 component-master generated from the same vector components contains criticals with up to at least 58 edges and 88 vertices;
- Components {0,±1} generate 332-1408 6D master which contains the 236-1216 list-master while originally components $\left\{0,\pm 1/2,\pm 1/\sqrt{3},\pm 1/\sqrt{2},1\right\}$ were used;
- In [37], we generated 6D criticals with up to 177 vertices and 87 edges from the list-master 236-1216, while, now, from the component-master 11808-314446, we obtain criticals with up to 201 vertices and 107 edges;
- We did not generate 16D and 32D masters because that would take too many CPU days and we already generated a huge number of criticals from submasters which are also defined by means of the same vector components in [37]. See also Section 3.

## 3. Methods

Our methods for obtaining quantum contextual sets boil down to algorithms and programs within the MMP language we developed to generate and handle KS MMP hypergraphs as the most elaborated and implemented kind of these sets. The programs we make use of, VECFIND, STATES01, MMPSTRIP, MMPSHUFFLE, SUBGRAPH, LOOP, SHORTD, etc., are freely available from our repository http://goo.gl/xbx8U2. They are developed in [5,29,30,38,39,40,47,48] and extended for the present elaboration. Each MMP hypergraph can be represented as a figure for a visualisation but more importantly as a string of ASCII characters with one line per hypergraph, enabling us to process millions of them simultaneously by inputting them into supercomputers and clusters. For the latter elaboration, we developed other dynamical programs specifically for a supercomputer or cluster, which enable piping of our files through our programs in order to parallelize jobs. The programs have the flexibility of handling practically unlimited number of MMP hypergraph vertices and edges as we can see from Table 2. The fact that we did not let our supercomputer run to generate 16D and 36D masters and our remark that it would be "computationally too demanding" do not mean that such runs are not feasible with the current computers, but that they would require too many CPU days on the supercomputer and that we decided not to burden it with such a task at the present stage of our research; see the explanation in Section 2.3.

## 4. Conclusions

The main result we obtain is that our vector component generation of KS hypergraphs (sets) exhaustively use all possible vectors that can be constructed from chosen vector components. This is in contrast to previous studies, which made use of serendipitously obtained lists of vectors curtailed in number due to various methods applied to obtain them. Hence, we obtain a thorough and maximally dense distribution of KS classes in all dimensions whose critical sets can therefore be much more effectively used for possible implementation in quantum computation and communication. A comparison of Table 1 and Table 2 vividly illustrates the difference.

**Figure A1:**
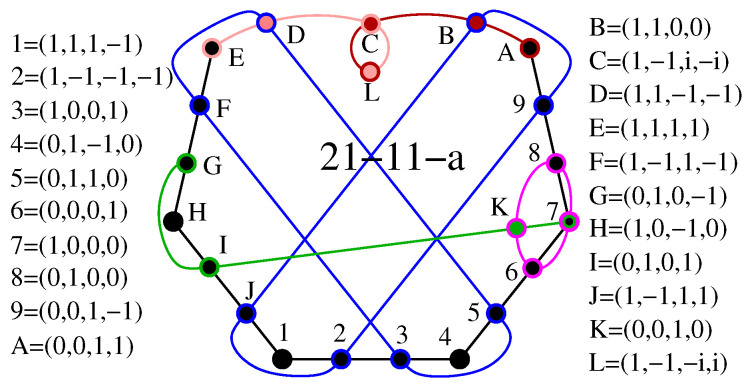
21-11 KS set with complex coordinatization.

In Appendix A, we present a possible experimental implementation of a KS critical with complex coordinatization generated from {0,±1,ω}. What we immediately notice about the 21-11 critical from Figure A1 is that the edges are interwoven in more intricate way than in the 18-9 (which has been implemented already in several experiments), exhibiting the so-called δ-feature of the edges forming the biggest loop within a KS hypergraph. The δ-feature refers to two neighbouring edges which share two vertices, i.e., intersect each other at two vertices [37]. It stems directly from the representation of KS configuration with MMP hypergraphs. Notice that the δ-feature precludes interpretation of practically any KS hypergraph in an even dimensional Hilbert space by means of so-called Greechie diagrams, which by definition require that two blocks (similar to hypergraph edges) do not share more than one atom (similar to a vertex) [6], on the one hand, and that the loops made by the blocks must be of order five or higher (which is hardly ever realised in even dimensional KS hypergraphs—see examples in [37]), on the other.

Our future engagement would be to tackle odd dimensional KS hypergraphs. Notice that, in a 3D Hilbert space, it is possible to explore similarities between Greechie diagrams and MMP hypergraphs because then neither of them can have edges/blocks which share more than one vertex/atom (via their respective definitions) and loops in both of them are of the order five or higher [26,39].

## Figures and Tables

**Figure A2:**
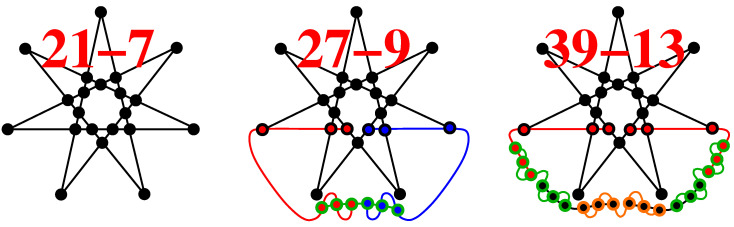
21-11 KS set from [19] and 27-9 are contained in three different master sets, 39-13 in two (together with 21-11 and 27-9); see the text.

**Table 1:** Vector lists from the literature; we call their masters *list-masters*. We shall make use of their vector components from the last column to generate master hypergraphs in Section 2.3 which we call *component-masters*. ω is a cubic root of unity: $\omega =e^{2\pi i/3}$.

dim	Master Size	Vector List	List Origin	Smallest Hypergraph	Vector Components
4D	24-24	[25,42,43]	symmetry, geometry	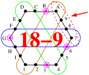	{0,±1}
4D	60-105	[28,37]	Pauli operators	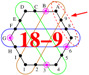	{0,±1,±i}
4D	60-75	[27,30,37,41]	regular polytope 600-cell	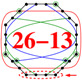	$\left\{0,\pm \left(\sqrt{5}-1\right)/2,\pm 1,\pm (\sqrt{5}+1)/2,2\right\}$
4D	148-265	[36,37]	Witting polytope	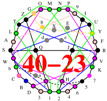	$\;\;\{0,\pm i,\pm 1,\pm \omega ,\pm \omega^{2},\pm i\omega^{1/\sqrt{3}},\pm i\omega^{2/\sqrt{3}}\}$
6D	21-7	[19]	symmetry	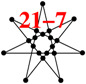	$\left\{0,1,\omega ,\omega^{2}\right\}$
6D	236-1216	Aravind & Waegell 2016, [37]	hypercube→hexaract Schäfli $\left\{4,3^{4}\right\}$	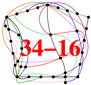	$\left\{0,\pm 1/2,\pm 1/\sqrt{3},\pm 1/\sqrt{2},1\right\}$
8D	36-9	[37]	symmetry	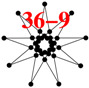	{0,±1}
8D	120-2025	[35,37]	Lie algebra E8	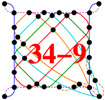	as given in [35]
16D	80-265	[37,44,45]	Qubit states	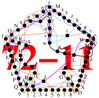	{0,±1}
32D	160-661	[37,46]	Qubit states	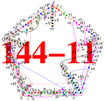	{0,±1}

**Table 2:** Component-masters we obtained. List-masters are given in Table 1. In the last two rows of all but the last column, we refer to the result [33] that there are 16D and 32D criticals with just nine edges. According to the conjectured feature (i) above, the masters generated by {0,±1} should contain those criticals; they did not come out in [37], so, we do not know how many vertices they have. The smallest ones we obtained are given in Table 1. The number of criticals given in the 4th column refer to the number of them we successfully generated although there are many more of them except in the 40-32 class.

dim	Vector Components	Component-Master Size	N^o^ of KS Criticals in Master	Smallest Hypergraph	Contains List-Masters
4D	{0,±1} or {0,±i} or $\left\{0,\pm \left(\sqrt{5}-1\right)/2\right\}$ or ...	40-32	6	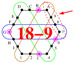	24-24
4D	{0,±1,±i}	156-249	$7.7\times 10^{6}$	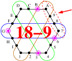	24-24, 60-105
4D	$\left\{0,\pm \left(\sqrt{5}-1\right)/2,\pm 1,\pm (\sqrt{5}+1)/2,2\right\}$	2316-3052	$1.5\times 10^{9}$	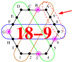	24-24, 60-75
4D	$\left\{0,\pm 1,\pm i,\pm \omega ,\pm \omega^{2}\right\}$	400-1012	$8\times 10^{6}$	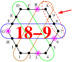	24-24, 60-105 148-265
6D	$\left\{0,\pm 1,\omega ,\omega^{2}\right\}$	11808-314446	$3\times 10^{7}$	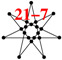	21-7, 236-1216
8D	{0,±1}	3280-1361376	$7\times 10^{6}$	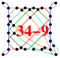	36-9, 120-2025
16D	{0,±1}	computationally too demanding	$4\times 10^{6}$	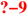 [33].	80-265
32D	{0,±1}	computationally too demanding	$2.5\times 10^{5}$	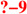 [33].	160-661

## Abbreviations

The following abbreviations are used in this manuscript:

KS	Kochen–Specker; defined in Section 1
MMP	McKay-Megill-Pavičić; defined in Section 2.1

## Appendix A. 21-11 KS Critical with Complex States from $H^{2}⊗H^{2}$

Below, we present a possible implementation of a KS critical 21-11 with complex coordinatization shown in Figure A1.

The vector components of the first qubit on a photon correspond to a linear (horizontal, *H*, vertical, *V*, diagonal, *D*, antidiagonal *A*) and circular (right, *R*, left *L*) polarization, and those of the second qubit to an angular momentum of the photon (+2,−2) and (h,v). One-to-one correspondence between them is given below.

An example of a tensor product of two vectors/states from $H^{2}⊗H^{2}$ is:

$$\begin{array}{c} |01\rangle =|0,1\rangle ={|0\rangle }_{1}⊗{|1\rangle }_{2}={\left(\begin{array}{c} 1\\ 0 \end{array}\right)}_{1}⊗{\left(\begin{array}{c} 0\\ 1 \end{array}\right)}_{2}=\left(\begin{array}{c} 1\left(\begin{array}{c} 0\\ 1 \end{array}\right)\\ 0\left(\begin{array}{c} 0\\ 1 \end{array}\right) \end{array}\right)=\left(\begin{array}{c} 0\\ 1\\ 0\\ 0 \end{array}\right). \end{array}$$

This is our vector 8 from Figure A1. Since we are interested in the qubit states, we are going to proceed in reverse—from 4-vectors to tensor products of polarization and angular momentum states. Let us first define them:

$$\begin{array}{c} |H\rangle ={\left(\begin{array}{c} 1\\ 0 \end{array}\right)}_{1};\;|V\rangle ={\left(\begin{array}{c} 0\\ 1 \end{array}\right)}_{1};\;|D\rangle =\frac{1}{\sqrt{2}}{\left(\begin{array}{c} 1\\ 1 \end{array}\right)}_{1};\;|A\rangle =\frac{1}{\sqrt{2}}{\left(\begin{array}{c} -1\\ 1 \end{array}\right)}_{1};\;|R\rangle =\frac{1}{\sqrt{2}}{\left(\begin{array}{c} 1\\ i \end{array}\right)}_{1};\; \end{array}$$

$$\begin{array}{c} |L\rangle =\frac{1}{\sqrt{2}}{\left(\begin{array}{c} 1\\ -i \end{array}\right)}_{1};\;|+2\rangle ={\left(\begin{array}{c} 1\\ 0 \end{array}\right)}_{2};\;|-2\rangle ={\left(\begin{array}{c} 0\\ 1 \end{array}\right)}_{2};\;|h\rangle =\frac{1}{\sqrt{2}}{\left(\begin{array}{c} 1\\ 1 \end{array}\right)}_{2};\;|v\rangle =\frac{1}{\sqrt{2}}{\left(\begin{array}{c} 1\\ -1 \end{array}\right)}_{2}. \end{array}$$

Now, one can read off our vertex states as follows:

$$\begin{array}{c} 1=\left(\begin{array}{c} 1\\ 1\\ 1\\ -1 \end{array}\right)\to \frac{1}{2}\left(\begin{array}{c} 1\\ 1\\ 1\\ -1 \end{array}\right)=\frac{1}{2}\left(\left(\begin{array}{c} 1\\ 1\\ 0\\ 0 \end{array}\right)+\left(\begin{array}{c} 0\\ 0\\ 1\\ -1 \end{array}\right)\right)=\frac{1}{\sqrt{2}}\left(\frac{1}{\sqrt{2}}\left(\begin{array}{c} 1\left(\begin{array}{c} 1\\ 1 \end{array}\right)\\ 0\left(\begin{array}{c} 1\\ 1 \end{array}\right) \end{array}\right)+\frac{1}{\sqrt{2}}\left(\begin{array}{c} 0\left(\begin{array}{c} 1\\ -1 \end{array}\right)\\ 1\left(\begin{array}{c} 1\\ -1 \end{array}\right) \end{array}\right)\right)\\ =\frac{1}{\sqrt{2}}({\left(\begin{array}{c} 1\\ 0 \end{array}\right)}_{1}⊗\frac{1}{\sqrt{2}}{\left(\begin{array}{c} 1\\ 1 \end{array}\right)}_{2}+{\left(\begin{array}{c} 0\\ 1 \end{array}\right)}_{1}⊗\frac{1}{\sqrt{2}}{\left(\begin{array}{c} 1\\ -1 \end{array}\right)}_{2}=\frac{1}{\sqrt{2}}(|H\rangle |h\rangle +\left|V\rangle |v\rangle \right)=\frac{1}{\sqrt{2}}(|D\rangle |+2\rangle -|A\rangle |-2\rangle ). \end{array}$$

We will now skip real states and go directly to those with imaginary components, *C* and *L*, to illustrate how they can be implemented via circular polarization:

$$\begin{array}{c} C=\left(\begin{array}{c} 1\\ -1\\ i\\ -i \end{array}\right)\to \frac{1}{2}\left(\begin{array}{c} 1\left(\begin{array}{c} 1\\ -1 \end{array}\right)\\ i\left(\begin{array}{c} 1\\ -1 \end{array}\right) \end{array}\right)=\frac{1}{\sqrt{2}}{\left(\begin{array}{c} 1\\ i \end{array}\right)}_{1}⊗\frac{1}{\sqrt{2}}{\left(\begin{array}{c} 1\\ -1 \end{array}\right)}_{2}=|R\rangle |v\rangle , \end{array}$$

$$\begin{array}{c} L=\left(\begin{array}{c} 1\\ -1\\ -i\\ i \end{array}\right)\to \frac{1}{2}\left(\begin{array}{c} 1\left(\begin{array}{c} 1\\ -1 \end{array}\right)\\ -i\left(\begin{array}{c} 1\\ -1 \end{array}\right) \end{array}\right)=\frac{1}{\sqrt{2}}{\left(\begin{array}{c} 1\\ -i \end{array}\right)}_{1}⊗\frac{1}{\sqrt{2}}{\left(\begin{array}{c} 1\\ -1 \end{array}\right)}_{2}=|L\rangle |v\rangle . \end{array}$$

Thus, in order to handle a complex coordinatization, we need a fifth degree of freedom (circular polarization), but, as we can see, it is manageable.

## Appendix B. 6D Criticals from the Masters Containing the Seven Context Star.

The 216-153 KS master generated from {0,1,ω} contains 21-7 and 27-9, which can be viewed as 21-7 with a pair of δ-triplets interwoven with 21-7, as shown in Figure A2. The 834-1609 KS master generated from $\{0,1,\omega ,\omega^{2}\},$ which were used for a construction of 21-7 in [19], contains 39-13 as well. Equally so, the 11808-314446 master generated from $\left\{0,\pm 1,\omega ,\omega^{2}\right\}$.
